# Investigating the flavor compounds in the cocoa powder production process

**DOI:** 10.1002/fsn3.1244

**Published:** 2019-11-22

**Authors:** Fariba Mohamadi Alasti, Narmela Asefi, Ramin Maleki, Seiied Sadegh SeiiedlouHeris

**Affiliations:** ^1^ Department of Food Science and Technology Tabriz Branch Islamic Azad University Tabriz Iran; ^2^ Research Department of Chromatography Iranian Academic Center for Education Culture & Research (ACECR) Urmia Branch Urmia Iran; ^3^ Department of Food Science and Technology Tabriz University Tabriz Iran

**Keywords:** Alkalization, cocoa powder, gas chromatography, mass spectrometry, roasting, volatile flavor compounds

## Abstract

Flavor is one of the most important quality properties of cacao beans, playing a key role in the admissibility of cocoa products, such as cocoa powder. This study examined the industrial processes influencing the flavor of cacao beans. The Ivory Coast cacao beans were used after their alkaline treatment with potassium carbonate (up to pH 7.5–8) and being roasted at 115–120°C for 60–70 min. The volatile components were extracted using Likens–Nickerson simultaneous distillation–extraction (SDE) apparatus. The volatile compound profiles were identified by means of gas chromatography–mass spectrometry (GC–MS), as a result of which several compounds (alcohols, carboxylic acids, aldehydes, ketones, esters, and pyrazines) were recognized. Alkalization and roasting were shown to be two important steps in the cacao beans processing that can affect the final cocoa powder flavor. In addition, pyrazines and esters were two major groups of flavor compounds formed during the roasting stage by the Maillard reaction. The percentage of 2,3,5,6‐tetramethylpyrazine was detected in the cacao beans equal to 0.5%. After the liquor pressing stage, tetramethylpyrazine increased to its highest amount (3%) in cocoa powder. It was found that the cocoa powder contained 2.69% of tetramethylpyrazine, 3.22% isobutyl benzoate, and 1.38% linalool. The highest percentage of increase in the mean amounts of 2,3,5,6‐tetramethylpyrazine, isobutyl benzoate, and linalool were observed in the roasting stage, after which the percentages diminished.

## Graphical abstract

This study examined the industrial processes influencing the flavor of cacao beans. Alkalization and roasting were shown to be two important steps in the cacao bean processing that can affect the final cocoa powder flavor. In addition, pyrazines and esters were two major groups of flavour compounds formed during the roasting stage by the Maillard reaction.

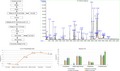

## 1 INTRODUCTION

Cocoa is a significant agricultural commodity, a cash product of great economic importance in the world, and the crucial raw material in chocolate and cocoa powder manufacturing. It is considered as the main agricultural export commodity for several cocoa–producing countries in West and Central Africa, such as Cote d'Ivoire, Nigeria, Cameroon, and Ghana (Afoakwa, Quao, Takrama, Budu, & Saalia, 2011). It is obtained from the beans of the *Theobroma cacao* (*T. cacao*) tree belonging to the *Malvaceae* family (Alverson, Whitlock, Nyfeller, Bayer, & Baum, 1999). *Theobroma cacao* is commercially cultivated in a climate belt within 20 degrees latitude of the equator (Bernaert, Blondeel, Allegaert, & Lohmueller, 2012; McShea et al., 2008). A cocoa pod is about 20 cm long and 10 cm wide (Owolarafe, Ogunsina, Gbadamosi, & Fabunmi, 2007), containing about 35 to 50 beans embedded in an amucilaginous pulp (Biehl & Ziegleder, 2003; McSheaet al., 2008). The pulp is proven to be rich in fermentable sugars molecules about 9 to 13% w/w (Lima, Almeida, Rob Nout, & Zwietering, 2011) such as glucose, fructose, and sucrose (Lefeber, Janssens, Camu, & Vuyst, 2010); high acidity (pH 3.0–3.5) due to the attendance of various organic acids, but mostly citric acid (Guehi, Zahouli, Ban‐Koffi, Fae, & Nemlin, 2010); and a protein content in the range of 0.4 to 0.6% w/w (Lima et al., 2011).

Several indexes are used to evaluate the quality of cacao beans, consisting of the bean size and count, bean color, and acidity of the bean. However, the most significant quality indexes of cacao beans are the quantity and kind of volatile flavor compounds (Magi, Bono, & Carro, 2012). Flavor is important in the admissibility of cacao beans and cocoa products, including chocolate (Afoakwa, Paterson, Fowler, & Ryan, 2008), consequently contributing to defining the quality of the products (Owusu, 2010). The special flavors of cacao beans are because of the rich volatile fractions composed of a mixture consisting of hundreds of compounds (Magi et al., 2012).

About 600 several compounds (alcohols, esters, aldehydes, ketones, carboxylic acids, and pyrazines) have been recognized as odor‐active compounds. The complex combination of cacao beans flavor depends on cacao beans genotype; postharvest acts (e.g., pulp preconditioning, fermentation, and drying); industrial processes such as roasting and alkalization; the class of soil; and the age of cacao trees. Cacao beans genotype influences both quantity and type of bean storage proteins, polyphenols, and carbohydrates. This determines the quantities and kinds of precursors created during the fermentation and drying processes effective on flavor formation, hence influencing both flavor type and intensity (Kongor, Hinneh, Walle, & Afoakwa, 2016). As an important attribute in admissibility of cocoa products, flavor is influenced not only by volatile aromatic compounds, but also by nonvolatile ones and the behavior of the continuous fat phase, influencing the distribution of volatiles into the mouth headspace and taste feeling. Precursor compound depends on the bean genotype, the environmental effects, and particularly the contents of stored proteins, polysaccharides, and polyphenols (Afoakwa et al., 2008; Schwan & Wheals, 2004).

To produce cocoa products, cocoa nibs are ground, roasted, and milled to obtain the cocoa liquor, which is then separated into cocoa powder and cocoa butter by pressing. If the production of the alkalized cocoa powder is intended, the comminuted cocoa nibs can be treated directly with sodium or potassium carbonate either on the fermented beans or on the cocoa mass after roasting.

Devised by van Houten, the alkaline treatment, known as “Dutching,” is mostly offered to partially omit acetic acid created during fermentation as well as develop a darker color and enhance the suspension of the cocoa particles in aqueous media. Cocoa powders are mainly applied in the preparation of milk‐based beverages for confectionery purposes (Bixler & Morgan, 1999). Researchers reported that the percentage of aromatic materials can be changed by changing the amount of sodium or potassium carbonate used during the alkalization process (Yue et al., 2012).

The previous literature showed that a number of researches have been conducted on cocoa alkalization and roasting process, but the changes of the flavor volatile compounds of cocoa occurring at different stages of the cocoa powder production process have not been investigated yet. The main aim of the present study was to study the changes of the flavor volatile compounds of cocoa in the process of producing cocoa powder at each stage (i.e., cacao beans, alkalization stage, roasting stage, milling stage, pressing stage, cocoa powder) at an industrial factory in Iran.

## 2 Materials and method

### 2.1 Chemicals

Potassium carbonate (K2CO3), hexane, and sodium sulfate (Na2SO_4_) were acquired from Sigma Chemical Co. (St. Louis). All the other HPLC grade solvents for the GC–MS analysis were procured from Sigma Chemical Co. (St. Louis, Missouri).

#### 2.1.1 Samples

**Figure 1:**
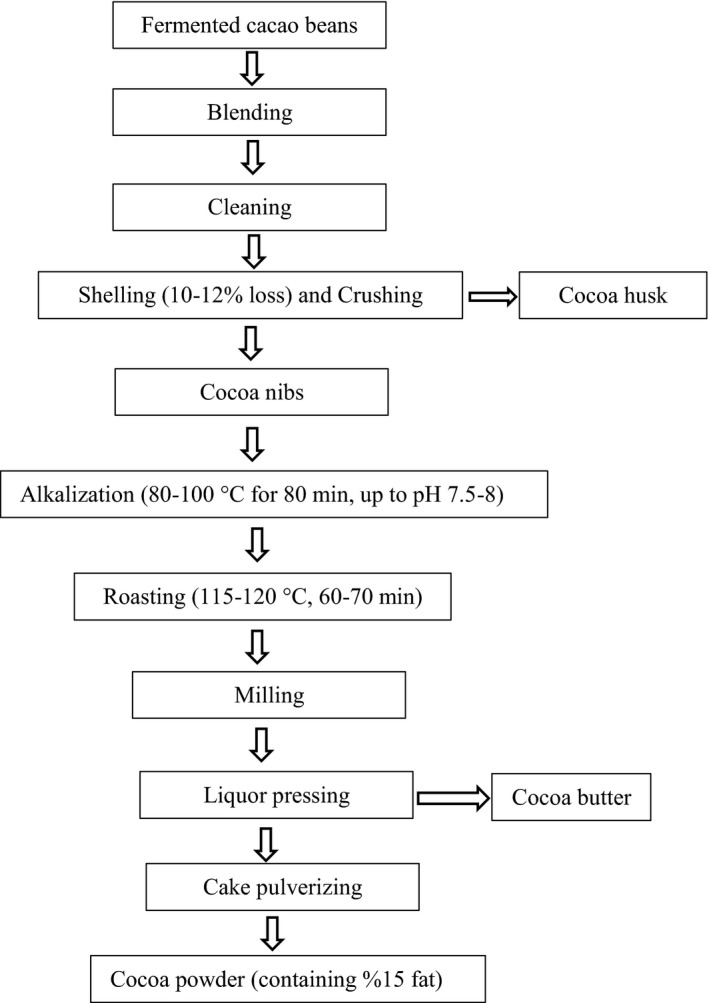
Diagram of the industrial cocoa powder production. Samples corresponding to a similar production group were collected from the fermented cacao beans, alkalized cacao beans, roasted cacao beans, milled beans, pressed liquor, and cocoa powder stages

The Ivory Coast cacao beans (*Forastero* cultivar, Ivory Coast) were used in the current study. The raw (fermented and dried) cacao beans were acquired from Ivory Coast by an industrial cocoa powder and chocolate producer in Tabriz, Iran. Since drying and fermentation accomplished in Ivory Coast were conformed to the local practices, there was no detailed data related to their quality. Cocoa powder production was carried out by a factory located in Tabriz, Iran. After the alkaline treatment with potassium carbonate (50 g/L), increasing the pH from 5.5 up to 7.5–8 at 80–100°C for 80 min, cacao beans were roasted at 115–120°C for 60–70 min. The milling was done under a two‐stage process: (a) Mill's stage in which the particle size reached 250–200 µm and (b) Stone mill stage in which the particle size reached 35–45 µ. The cocoa liquor was exited from containers and pressed (by hydraulic press) and converted to the cocoa powder and cocoa butter. The samples obtained from the raw cacao beans, alkalized cacao beans, roasted cacao beans, milled cacao beans, pressed liquor, and cocoa powder were classified into three groups in order to find out the effects of the production process on the cocoa powder flavor. A diagram of the stages involved in cocoa powder production is shown in Figure 1. The cacao beans and other samples were milled and pulverized by laboratory mill (M 20 Universal mill; IKA) at room temperature (27ºC) for 45s to obtain homogeneous samples. The liquor was homogenized by a mixer (CJJ‐2/ 2A Series).

#### 2.1.2 Extraction of the volatile components

Twenty‐five grams of the pulverized samples were added to the distilled water (100 ml) and heated for 60 min using Likens–Nickerson simultaneous distillation–extraction (SDE) apparatus (Schultz, Flath, Mon, Eggling, & Terranishi, 1977). The volatiles were trapped in hexane (2 ml). The extract was dried over 5 g of anhydrous Na_2_SO_4_ and localized on a Vigreux column (Sigma Aldrich). Finally, one microliter of each volatile component was analyzed on the GC–MS.

#### 2.1.3 GC‐MS conditions

The aromatic compounds were identified and confirmed by GC‐MS. For the purpose of GC–MS analysis, an Agilent 7890 A gas chromatograph coupled to a 5975A mass spectrometer using a HP‐5 MS capillary column (5% phenyl methylpolysiloxane, 30 m length, 0.25 mm i.d., 0.25 μm film thickness) was used. The oven temperature was planned as follows: 3 min at 80°C, subsequently 8°C min −1 to 180°C, and retained for 10 min at 180°C. Helium was applied as carrier gas at the flow speed of 1 ml/min, and electron impact (EI) was 70 eV. The injector was set in a split mode (split ratio of 1:500), and mass range acquisition was from 40 to 500 m/z. The main oil compounds were identified by utilizing the calculated linear retention indicators (NIST, 2005; Wiley, 2007) and mass spectra with those offered in the NIST 05 and Wiley 07.

### 2.2 Statistical analysis

Statistics on a completely randomized design (CRD) were performed by the analysis of variance (ANOVA) procedure, using Mstatc 11 software (Michigan State University). The Duncan test was applied to compare the difference among mean values at a significant level of 0.01 (*p* ≤ .01). All experiments were carried out in three replications.

## 3 RESULTS AND DISCUSSION

**Figure 2:**
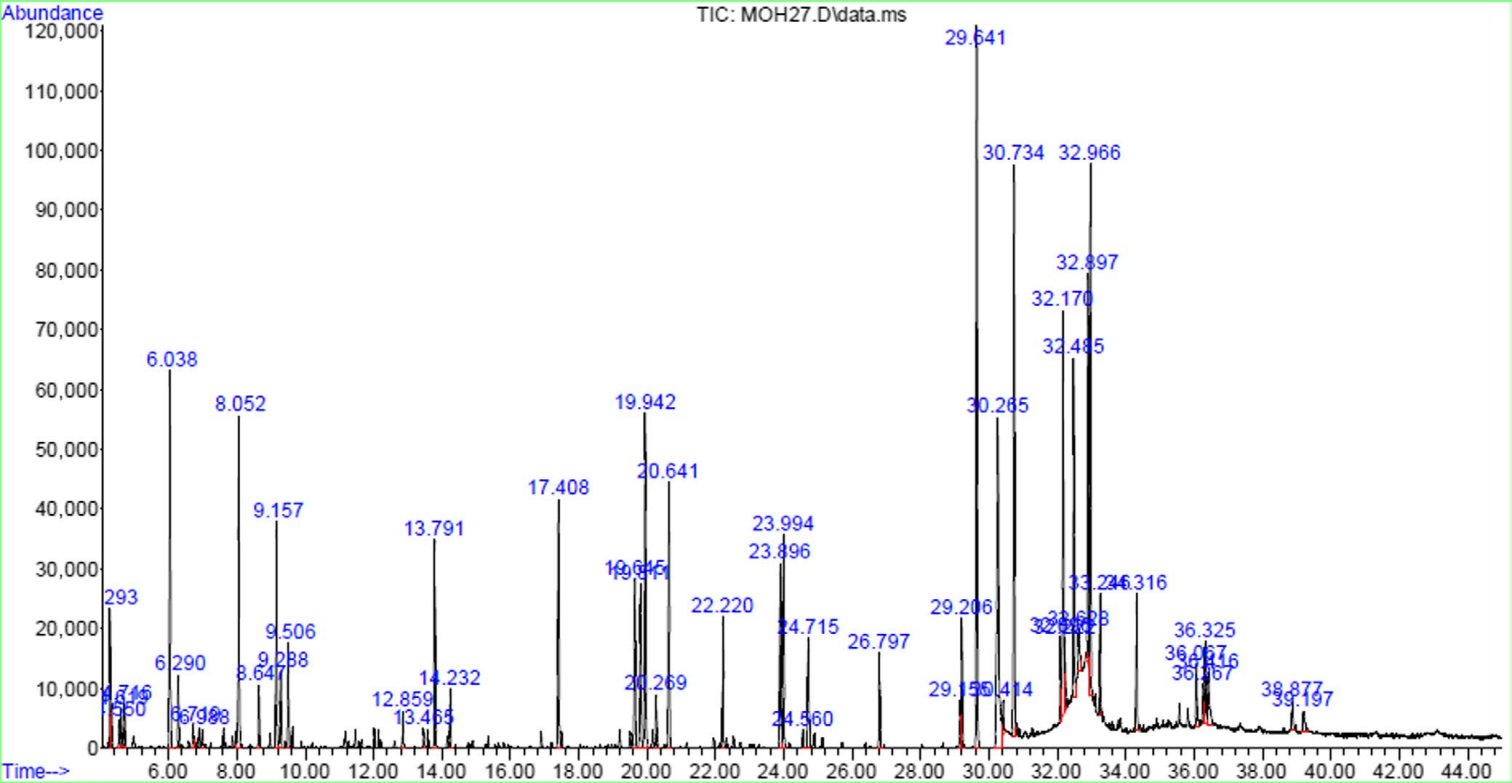
Cocoa powder chromatogram of the aromas obtained using gas chromatography–mass spectrometry

Some of the chemical properties of the cacao beans are shown in Table 1. Tables 2 and 3 show a number of the volatile compounds found at each stage in the processing of cacao beans to obtain cocoa powder. GC‐MS identified many volatile aromatic components in the cocoa powder and other samples. A typical chromatogram of cocoa powder is shown in Figure 2.

**Table 1:** The chemical characteristics of the cacao bean (w/w %)*

Sample	Moisture (%)	Protein (%)	Fat (%)	Ash (%)	Glucose (%)	pH	Titratable acidity (acetic acid (%))
The Ivory Coas cacao bean	3.46	14.37	55.79	6.63	1.58	5.64	0.22

* Numbers are the average of three replicate.

**Table 2:** Main cocoa on‐odor volatile compounds of samples in different stages of production*

Flavor compounds	R.T†	Content of volatile compounds in total volatile flavor components (w/w %)						
		Cacao Bean	Alkalization stage	Roasting stage	Milling stage	Pressing stage	Cocoa powder	Odor quality‡
Alcohols								
Linalool	9.49	0.05 ± 0.01^C^	0.50 ± 0.15^B^	1.05 ± 0.20^A^	1.09 ± 0.10^A^	1.13 ± 0.13^A^	1.38 ± 0.18^A^	Floral, green
2‐Hexanol	4.61	0.03 ± 0.02^A^	0.03 ± 0.02^A^	0.05 ± 0.04^A^	0.05 ± 0.04^A^	0.06 ± 0.04^A^	0.05 ± 0.01^A^	Fruity, green
1‐Hexanol	4.55	0.02 ± 0.01^A^	0.03 ± 0.02^A^	0.05 ± 0.04^A^	0.06 ± 0.04^A^	0.06 ± 0.04^A^	0.04 ± 0.02^A^	Fruity, green
Aldehydes								
Benzaldehyde	6.04	0.38 ± 0.16^D^	0.56 ± 0.20^D^	0.80 ± 0.32^CD^	1.40 ± 0.20^BC^	1.70 ± 0.20^B^	4.67 ± 0.33^A^	Nutty,almond‐like
Phenylacetaldehyde	8.05	0.10 ± 0.02^D^	1.10 ± 0.10^C^	2.02 ± 0.20^B^	2.17 ± 0.17^B^	3.97 ± 0.40^A^	4.21 ± 0.30^A^	Honey‐floral
5‐methyl −2‐Phenyl −2‐hexenal	19.81	–	–	0.42 ± 0.30^B^	0.96 ± 0.30^B^	2.68 ± 0.10^A^	2.16 ± 0.30^A^	Cocoa
2‐hexenal	36.06	0.16 ± 0.05^C^	–	0.40 ± 0.25^BC^	1.30 ± 0.20^A^	0.70 ± 0.30^BC^	0.92 ± 0.35^AB^	Fruity, green
Esters								
Isoamyl acetate	4.29	0.23 ± 0.10^B^	–	1.66 ± 0.10^A^	1.60 ± 0.20^A^	1.23 ± 0.23^AB^	1.53 ± 0.23^A^	Fruity, banana
2‐Phenethyl acetate	13.78	0.17 ± 0.10^C^	0.18 ± 0.10^C^	0.55 ± 0.67^C^	1.70 ± 0.02^B^	2.50 ± 0.15^A^	2.68 ± 0.20^A^	Floral, honey
Isobutyl benzoate	17.40	0.38 ± 0.07^B^	–	3.30 ± 0.30^A^	3.50 ± 0.25^A^	3.60 ± 0.20^A^	3.22 ± 0.22^A^	Balsam, sweet
Ethyl laurate	22.21	0.07 ± 0.02^C^	–	1.10 ± 0.10^B^	1.28 ± 0.28^AB^	1.06 ± 0.06^B^	1.62 ± 0.20^A^	Fruity, floral
Ketones								
2‐Heptanone	4.59	–	–	0.38 ± 0.10^A^	0.28 ± 0.15^A^	0.27 ± 0.10^A^	0.26 ± 0.20^A^	Fruity, floral
Acetophenon	8.64	–	–	0.53 ± 0.21^A^	0.50 ± 0.15^A^	0.78 ± 0.20^A^	0.77 ± 0.20^A^	Floral
Methyl heptyl ketone	9.28	0.14 ± 0.07^B^	–	0.97 ± 0.40^A^	0.95 ± 0.05^A^	1.0 ± 0.10^A^	0.90 ± 0.30^A^	Fruity, green
2‐Pentadecanone	29.30	–	–	1.13 ± 0.15^B^	0.98 ± 0.20^B^	1.04 ± 0.20^B^	1.52 ± 0.20^A^	Floral
Pyrazines								
2‐Ethyl−3‐methylpyrazine	6.90	–	0.21 ± 0.20^B^	0.23 ± 0.10^B^	0.25 ± 0.20^A^	0.31 ± 0.10^A^	0.26 ± 0.20^A^	Nutty, cocoa
2,3,5,6 Tetramethylpyrazine	9.15	0.50 ± 0.10^C^	o.30 ± 0.20^C^	1.96 ± 0.20^B^	2.90 ± 0.20^A^	3.00 ± 0.10^A^	2.69 ± 0.20^A^	Chocolate, coffee, cocoa
2,5‐Dimethylpyrazine	13.46	–	–	0.30 ± 0.20^A^	0.31 ± 0.10^A^	0.37 ± 0.17^A^	0.23 ± 0.13^A^	Cocoa, rusted nuts
2,3,5‐trimethyl pyrazine	14.32	0.05 ± 0.04^B^	–	0.09 ± 0.04^B^	0.73 ± 0.20^A^	0.74 ± 0.20^A^	0.66 ± 0.33^A^	Cocoa, rusted nuts, peanut
Pyrrole								
1H‐Pyrrole, 1‐pentyl	8.31	–	0.62 ± 0.20	–	–	–	–	Green
Indole (1H‐Indole)	14.77	–	0.52 ± 0.20^A^	–	–	0.29 ± 0.20^A^	–	Chocolate, green

Mean values with different superscript letters are significantly different (*p* ≤ .01).

* Values are mean ± *SD* of three separate determinations.

^†^ Retention time (min).

^‡^ Aprotosoaie et al., 2016 & Website: http://www.chemspider.com.

**Table 3:** Main off‐odor volatile compounds in cocoa flavor of samples in different stages of production*

Off‐odor volatile Compounds	R.T†	Content of off‐odor volatile compounds in total volatile flavor components (w/w %)						
		Cacao Bean	Alkalization stage	Roasting stage	Milling stage	Pressing stage	Cocoa powder	Odor quality‡
Alcohols								
2‐Decanol	24.56	0.05 ± 0.02^B^	0.07 ± 0.01^B^	0.89 ± 0.10^A^	0.09 ± 0.01^B^	1.00 ± 0.30^A^	0.09 ± 0.02^B^	Fatty, waxy
Esters								
Ethyl myristate	26.79	0.08 ± 0.02^B^	–	1.30 ± 0.20^A^	1.40 ± 0.20^A^	1.23 ± 0.20^A^	1.43 ± 0.20^A^	Waxy, soapy
Methyl palmitate	30.73	0.52 ± 0.20^D^	0.57 ± 0.30^D^	1.20 ± 0.15^C^	3.30 ± 0.10^B^	4.06 ± 0.35^A^	4.20 ± 0.10^A^	Oily, waxy
Methyl stearate	32.47	0.38 ± 0.01^B^	–	3.30 ± 0.20^A^	3.50 ± 0.20^A^	3.60 ± 0.20^A^	3.22 ± 0.22^A^	Oily
Ethyl Oleate	32.96	–	0.43 ± 0.10^D^	1.50 ± 0.20^C^	4.70 ± 0.20^B^	4.80 ± 0.20^B^	5.20 ± 0.10^A^	Waxy, dairy
Acids								
Oleic acid	32.66	13.23 ± 1.00^A^	0.66 ± 0.10^D^	3.83 ± 0.10^B^	3.45 ± 0.45^BC^	2.38 ± 0.20^C^	0.66 ± 0.20^D^	Lard‐like
Palmitic acid	29.64	15.89 ± 1.00^A^	1.93 ± 0.20^C^	7.20 ± 1.00^B^	6.80 ± 1.00^B^	6.70 ± 0.20^B^	7.10 ± 0.10^B^	Fatty, waxy
Stearic acid	32.89	5.01 ± 0.20^A^	0.66 ± 0.20^D^	4.04 ± 0.20^B^	3.50 ± 0.20^C^	3.60 ± 0.20^BC^	3.80 ± 0.20^BC^	Fatty, tallow
Alkanes								
Nonane	4.71	0.07 ± 0.01^BC^	0.03 ± 0.02^C^	0.30 ± 0.20^AB^	0.35 ± 0.20^A^	0.33 ± 0.21^A^	0.40 ± 0.20^A^	Gasoline‐like
Nonadecane	29.15	7.54 ± 0.20^B^	10.46 ± 1.00^A^	6.47 ± 0.20^B^	4.52 ± 0.20^C^	0.97 ± 0.22^D^	0.61 ± 0.20^D^	Odorless
Tricosane	34.32	–	–	0.30 ± 0.20^A^	0.34 ± 0.15^A^	0.37 ± 0.02^A^	0.36 ± 0.02^A^	Waxy
Heptacosane	36.31	2.84 ± 0.22^A^	0.91 ± 0.20^BC^	0.95 ± 0.20^BC^	1.40 ± 0.20^B^	0.63 ± 0.02^C^	0.94 ± 0.20^BC^	Odorless
Pentacosane	36.27	9.37 ± 0.53^B^	13.12 ± 0.37^A^	7.60 ± 0.47^B^	4.20 ± 0.51^C^	1.40 ± 0.20^CD^	0.54 ± 0.20^D^	–

Note

Mean values with different superscript letters are significantly different (*p* ≤ .01).

* Values are mean ± *SD* of three separate determinations.

^†^ Retention time (min).

^‡^ Aprotosoaie et al., 2016 & Website: http://www.chemspider.com.

Many volatile aromatic materials included alcohols, acids, aldehydes, esters, ketones, pyrazines, and pyrrole compounds (Table 2), which, according to previous studies, are the most effective compounds in creating the cocoa products’ flavor. Therefore, the investigation on the production stages was focused on these compounds. Although the same compounds were identified by earlier studies conducted on chocolate and other cocoa products, disagreements still existed in the reported number of volatile flavor compounds recognized in these products. Owusu (2010) found more pyrazine‐type compounds, for example, in dark chocolate, compared to what was found in the present study. Such differences may be originated from the type of the cocoa, the fermentation/drying technique, and the industrial process. In the case of alkalized cocoa powder, the most significant processes were the degree of roasting temperature to which the cacao beans are exposed and the varying degrees of alkalization.

### 3.1 Pyrazines

Table 2 shows a number of pyrazine compounds present in the samples. Compounds such as 2‐ethyl‐3‐methylpyrazine, 2,5‐dimethylpyrazine, 2,3,5‐trimethylpyrazine, and 2,3,5,6 tetramethylpyrazine were detected in the samples. Most of the pyrazine compounds in the current study were formed during the roasting stage and maintained in the subsequent processes, thus found in cocoa powder compounds. 2,3,5,6‐tetramethylpyrazine was detected in the cacao beans (0.5%) and increased to 2.69% in the cocoa powder.

Pyrazine compounds are the key type of heterocyclic volatiles and the main components forming the cocoa aroma. They exhibit nutty, roasty, green, and earthy aromas (Czerny et al., 2008; Czerny & Grosch, 2000). Tetramethylpyrazine and trimethylpyrazine are the most important pyrazines. Trimethylpyrazine displays nutty, grassy, and constant cocoa notes, while tetramethylpyrazine gives the gravy properties to the cocoa flavor (Ramli, Hassan, Said, Samsudin, & Idris, 2006). According to the present data, the 2,3,5‐trimethylpyrazine and 2,3,5,6‐tetramethylpyrazine are the two naturally occurring pyrazines created in considerable amounts in the unroasted nib (Hashim & Chaveron, 1994). However, our results showed that 2,3,5,6‐tetramethylpyrazine was the major pyrazine present in the cacao beans. These results were in line with those obtained by Reineccius, Keeney, and Weissberger (1972), where it was reported that only 2,3,5,6‐tetramethylpyrazine was present in the unroasted beans. 2‐Ethyl‐3‐methylpyrazine and 2,5‐dimethylpyrazine were not detected in the cacao beans.

**Figure 3:**
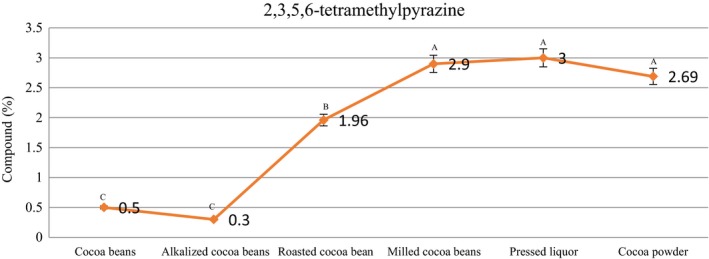
Content of 2,3,5,6 tetramethylpyrazine in the samples

Żyżelewicz et al. (2018) stated that the pyrazine compounds were created in higher temperatures in the roasting stage and may be an indicator of cacao beans’ roasting. Most of the pyrazine is formed from the *α*‐aminoketones through Strecker degradation and Maillard reactions during roasting (Rodriguez‐Campos, Escalona‐Buendia, Orozco‐Avila, Lugo‐Cervantes, & Jaramillo‐Flores, 2011). Tetramethylpyrazine could be formed during fermentation as a metabolic product of *Bacillus subtilis* (Ramli et al., 2006). Considering the fact that parazines are produced in the roasting process, while the roasting temperature of this study was 115°C, most of the parazines were detected after this stage. The highest percentage of increase in the amount of tetramethylpyrazine was observed in the roasting stage (growth 84.69%), and then, the percentage of increase in the amount of tetramethylpyrazine was observed in the milling stage (growth 32.41%). In the other production stages, no percentage of increase in the amount of tetramethylpyrazine was observed (Figure 3).

The cacao beans type and the fermentation stage are critical factors affecting the creation of pyrazines, and our results indicated a low level of pyrazines present in the Ivory Coast cacao beans.

### 3.2 Esters

As can be seen in Table 2, during the stage of alkalization, some ester compounds in the cacao beans, such as isoamyl acetate, isobutyl benzoate, and ethyl laurate, disappeared, while they appeared in the roasting stage. This indicated that they were formed during the Maillard reaction in the roasting stage and maintained in subsequent processes, and these esters were found in cocoa powder compounds. Isobutyl benzoate was found in the highest amount (3.22%), compared to other esters in cocoa powder.

Esters are the second most significant kind of volatiles after pyrazines. Ethyl‐, methylesters, and acetates prevail in this class (Ramli et al., 2006; Rodriguez‐Campos et al., 2011). Displaying a fruity flavor, they are the generic aroma components (mostly acetates) in the unroasted cocoas and are derived from amino acids (Biehl & Ziegleder, 2003). 2‐Phenylethylacetate has flowery and honey notes (Jinap, WanRosli, Russly, & Nurdin, 1998).

**Figure 4:**
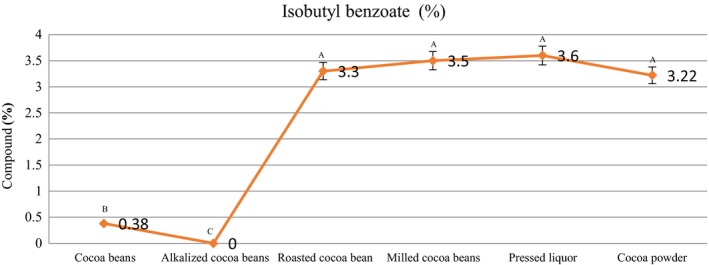
Content of isobutyl benzoate in the samples

Table 2 shows a number of esters present in the samples with desirable flavors. A summary of the main odor‐damaging compounds to the cocoa aroma is presented in Table 3. There were esters that can be produced by the decomposition of fatty acids, and their flavors were undesirably waxy (Table 3). High amounts of 2‐phenylethylacetate and low quantities of 3‐methyl‐1‐butanol acetate were important factors for the flavor property of cocoa (Rodriguez‐Campos et al., 2012). Our results showed that in this study, high amounts of 2‐phenylethylacetate could be detected, while 3‐methyl‐1‐butanol was not detected. The highest increase in the percentage of the isobutyl benzoate amount was observed in the roasting stage (growth 100%), and another increase was observed in the milling stage (growth 2.7%). In the next production stages, it was not seen any increase in the percentage of the isobutyl benzoate amount (Figure 4).

High temperatures during roasting negatively affect the content of esthers (Ramli et al., 2006), while Jinap et al. (1998) reported that the highest unit area of esters was obtained in nib roasted at higher temperatures (160–170°C) but at shorter time (5–15 min), Keeney (1972) reported that higher temperatures (150–170°C) with longer roasting time (45–65 min) might cause the destruction of esters. In this study, the nibs were roasted at relatively low temperature (115–120°C).

### 3.3 Aldehydes and ketones

**Figure 5:**
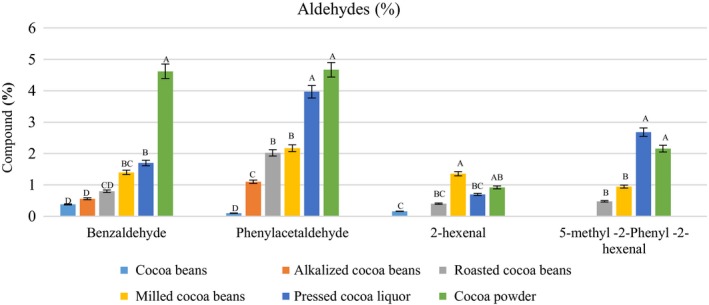
Content of aldehydes in the samples

Table 2 and Figure 5 show a number of aldehydes detected in the samples as well as the percentage at which they were measured. In our study, compounds such as benzaldehyde, phenylacetaldehyde, and 2‐hexenal were detected in the cacao beans, but 5‐methyl‐2‐phenyl‐2‐hexenal was not found in the cacao beans and was formed via the Maillard reaction after roasting. The carbonylic compounds of the aldehyde form are critical for the development of a good cocoa aroma. A great amount of aldehydes as well as ketones is desirable for cocoa characteristics (Rodriguez‐Campos et al., 2012). Usually, they are derived from Strecker degradation of free amino acids during roasting and are vital for the development of the cocoa aroma (Aprotosoaie, Luca, & Miron, 2016). However, low amounts of aldehydes may be formed even during fermentation and drying stages (Rodriguez‐Campos et al., 2012). 5‐Methyl‐2‐phenyl‐2‐hexenal displays a deep bitter cocoa note (Ramli et al., 2006). High temperatures and a longer roasting period reduce the quantity of aldehydes (pyrazines & Ziegleder, 2009). In the current study, roasting temperature was relatively low, so the content of aldehydes was found to be optimal. Jinap et al (1998) reported that carbonyls were present in the highest unit area at a lower temperature (110–120°C) with a longer roasting time (55–65 min). Not only are aldehydes flavor components, but they also are significant reactants interfered in the creation of heterocyclic compounds (e.g., pyrazines; Ziegleder, 2009). In this study, 2‐phenylacetaldehyde and benzaldehyde were found in high concentrations (Figure 5, respectively, 4.21%, 4.67%). 2‐Phenylacetaldehyde formed by Strecker degradation of phenylalanine was present in much higher amounts after roasting (Aprotosoaie et al., 2016).

**Figure 6:**
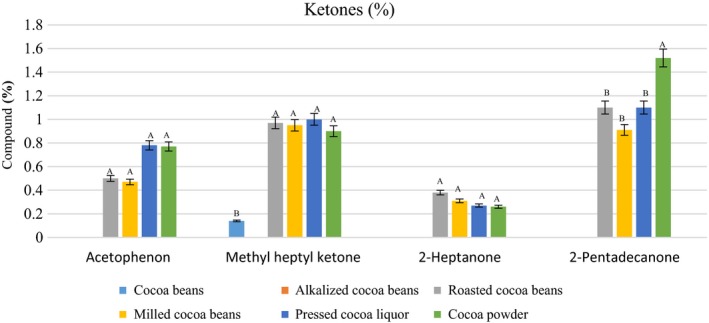
Content of ketones in the samples

Table 2 and Figure 6 show a number of ketones compounds present in the samples. Compounds such as 2‐heptanone, acetophenon, and 2‐pentadecanone were not detected in the cacao beans and were formed by the Maillard reaction after roasting, but 2‐nonanone was detected in the cacao beans. Most of the ketones compounds in our study were formed during the roasting stage and maintained in the subsequent processes, thus found in cocoa powder compounds. Among the ketones, acetophenone created sweet, floral notes (Rodriguez‐Campos et al., 2012).

### 3.4 Alcohols

**Figure 7:**
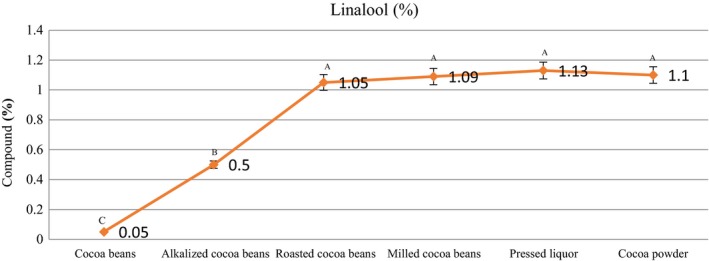
Content of linalol in the samples

Table 2 shows a number of alcoholic compounds present in the samples. Table 3 shows an alcoholic compound (2‐decanol) present in the samples, displaying aroma‐damaging characteristics, and found to be in very low levels. Among the alcoholic compounds, the amount of linalool was found to be significant. Linalool and 2‐phenylethanol are the main alcohols in roasted nibs (Jinap et al., 1998). 2‐Phenylethanol was not observed in our samples. The ratio of linalool/benzaldehyde may be applied as a flavor indicator; a quantity over 0.3 shows typical fine‐grade cocoas (Ziegleder, 2009). In our study, the flavor indicator value was about 0.3. Jinap et al (1998) reported that the quantity of alcohols reduced as both roasting temperature and roasting time increased, attributable to either the volatilization or destruction of alcohols during roasting. However, this decrease was not observed in our study due to the fact that the roasting temperature in our study was relatively low. In our study, a number of alcoholic compounds in low amounts were found in the cacao beans formed via the Maillard reaction after roasting and maintained in the subsequent processes, and found in cocoa powder compounds. Among the alcoholic compounds, the amount of linalool was significant at the level (1.38%, Figure 7).

Alcohols are assumed to be produced from microbial activity during the fermentation of beans (Aprotosoaie et al., 2016). In addition, they may be produced from heat depreciation of amino acids. During drying and roasting, the amount of alcohols reduces through chemical degradation or volatilization. High temperatures (160°C to 170°C) and extended heat duration increase the percentage of loss for alcohols (Ramli et al., 2006).

Alcohols exhibit a floral, fruity, and green flavor. High alcohol concentrations are favorable in order to obtain cocoa products with candy and flowery notes (Rodriguez‐Campos et al., 2012). The linalool shows green and floral aromas (Bonvechi, 2005). Linalool is derived from the monoterpenes class (Flament, 1991).

Although similar alcoholic compounds have been reported by earlier researchers in chocolate and other cocoa products, disagreements exist in the reported number of alcoholic compounds found in these products.

### 3.5 Phenols

Phenols (phenol, 2‐methoxyphenol) are compounds with aroma‐damaging properties, creating smoky and unwanted notes (Jinap et al., 1998). High quantities of phenols perhaps result from the wood fire smoke during drying (Ziegleder & Biehl, 1988). Smoke from wood or charcoal fires can also contaminate cocoa drying (Lehrian, Keeney, & Lopez, 1978). Indeed, a high‐quality cocoa should be typically free of them (Jinap et al., 1998).

In this study, roasting temperature was relatively low, so phenolic compounds were not detected.

### 3.6 Other components

Table 2 shows a number of pyrrole compounds present in the samples. Pyrrole compounds are formed in drying and roasting stages via Strecker degradation and Maillard reactions starting from the amino acid proline. They display desirable caramel, chocolate, and roasty notes (Rodriguez‐Campos et al., 2012).

A summary of the major compounds damaging the cocoa flavor found in the samples are provided in Table 3. Among them, the number and amount of alkanes were relatively high.

Alkanes existed in very high amounts in the cacao beans and were maintained in the subsequent processes and found in cocoa powder compounds. Their flavor was undesirably waxy and gasoline‐like.

## 4 CONCLUSIONS

Cocoa flavor is unique, mixed, and deceptive. Volatile chemical components contribute to the cocoa aroma. Many physical, chemical, and biological factors influence the formation and the development of flavor. The formation of flavor precursors during fermentation is essential for the development of the final flavor. The roasting and alkalization stages are important variables which affect the development of the cocoa flavor. Cacao beans alkalization is one of the important factors of Maillard reaction with the alteration of pH and also advances an interaction between polysaccharides, proteins, polyphenols, and Maillard products.

Many volatile compounds were detected in samples. Among them, pyrazines and esters were two major groups which existed in the cocoa volatiles.

To produce cocoa powder with a perfect flavor, cacao beans including lower off‐odor volatile compounds such as alkane compounds should be selected.

For the production of high‐quality cocoa powder, further investigation of the industrial processes contributing to the variations in the aroma character, especially the alkalization and roasting stages, seems necessary.

## CONFLICT OF INTEREST

The authors declare that they do not have any conflict of interest.

## ETHICAL APPROVAL

This study does not involve any human or animal testing.
